# Prediction Model of Lymph Node Metastasis Risk in Elderly Patients with Early Gastric Cancer before Endoscopic Resection: A Retrospective Analysis Based on International Multicenter Data

**DOI:** 10.7150/jca.56702

**Published:** 2021-07-25

**Authors:** Siwei Pan, Wen An, Yuen Tan, Qingchuan Chen, Pengfei Liu, Huimian Xu

**Affiliations:** 1 Department of Surgical Oncology, The First Affiliated Hospital of China Medical University, Shenyang 110001, Liaoning, China; 2 Department of Surgical Oncology and General Surgery, Key Laboratory of Precision Diagnosis and Treatment of Gastrointestinal Tumors, Ministry of Education, The First Affiliated Hospital of China Medical University, Shenyang, China

**Keywords:** Early gastric cancer, lymph node metastasis, elderly patients, nomogram

## Abstract

**Background:** Patients with early gastric cancer (EGC) must suffer reoperation if diagnosed with a high possibility of lymph node (LN) metastasis. The purpose of the current study was to develop and validate a model to predict the risk of LN metastasis in elderly patients before endoscopic resection.

**Methods:** A total of 1911 EGC patients who had undergone radical surgery were selected and assigned randomly (2:1) to either the training cohort or the validation cohort. A nomogram was established based on the univariate and multivariate logistic regression models using the training cohort. Cox proportional hazards regression models were applied to identify the prognostic factors in univariate and multivariable analyses.

**Results:** Three variables—tumor size, grade, and T stage—were derived from the multivariate analyses in the training cohort and incorporated into the nomogram. The AUC of the nomogram was 0.732 in the training cohort and 0.706 in the validation cohort. There were significant differences in survival among patients with different degrees of LN metastasis risk (training cohort: five-year disease-specific survival (DSS): low risk 88.1% vs. moderate risk 80.0% vs. high risk 72.9%, P < 0.001; validation cohort five-year DSS: low risk 89.0% vs. moderate risk 84.3% vs. high risk 72.2%, P < 0.001). The LN metastasis risk assessed from the model was also an independent prognostic factor.

**Conclusion:** We established a nomogram that accurately predicts LN metastasis risk for elderly patients with EGC before endoscopic resection to avoid further injury from reoperation.

## Introduction

Early gastric cancer (EGC) in which tumor invasion is restricted to the mucosa (T1a) or submucosa (T1b), regardless of lymph node (LN) metastasis status, has a significantly more favorable prognosis than advanced gastric cancer (GC), and has an outstanding five-year survival rate following radical resection 1, 2. Currently, endoscopic submucosal dissection (ESD) and endoscopic mucosal resection (EMR) have been recognized as standard treatments that can achieve en bloc resection of EGC 3, 4. However, the above treatments do not effectively dissect perigastric LNs. Patients could also require additional radical gastrectomy after diagnosis of high-risk LN metastasis following postoperative pathological examination 5-7. The LN metastasis rate has been reported in previous studies to range from 8.5% to 20.1% 8-10.

Since LN metastasis is recognized as an independent prognostic factor for GC, the occurrence of LN metastasis will greatly increase the risk of postoperative recurrence and shorten postoperative survival time in EGC 2, 11, 12. It was reported that EGC patients with metastasis LNs (mLNs) had more than five times the risk of postoperative recurrence than EGC patients without metastasis 11. Therefore, it is of great significance to evaluate the risk of LN metastasis in advance of treatment decisions.

To date, several studies have developed different prediction models. The eCura system proposed by the Japanese Gastric Cancer Association (JGCA) is more authoritative and is included in the fifth edition of the Japanese Gastric Cancer Treatment Guideline 13, 14. It evaluates LN metastasis risk of post-ESD patients based on five factors: lymphatic invasion, tumor size, vertical margin, venous invasion, and degree of submucosal invasion. Further, it suggests that patients with intermediate and high risk undergo additional radical gastrectomy. Nevertheless, the shortcoming of this system is that it is evaluated after ESD. Additional surgery might be a severe blow to a patient's body, especially for patients at an advanced age. Thus, a preoperative risk assessment model that can rapidly analyze LN metastasis risk based on clinical data is particularly important for elderly patients.

Hence, in the current study, we aimed to construct a preoperative LN risk assessment model for elderly patients with EGC. We used the First Affiliated Hospital of China Medical University (CMU) and the Surveillance, Epidemiology, and End Results (SEER) databases to identify which group was suitable for direct radical gastrectomy.

## Materials and Methods

### Patient Source

The SEER program has collected and published incidence and survival data based on cancer registries, and covers approximately 26% of the US population 15, 16. The cohort for the current study was selected from the SEER database using SEER-stat software (SEER*Stat 8.3.6). We obtained permission to access research data files with the reference number 10944-Nov2019.

Patients who had undergone radical gastrectomy at the Department of Surgical Oncology, the First Hospital of CMU (Shenyang, China) from January 1980 to December 2012, and patients who had undergone gastrectomy and were subsequently diagnosed with gastric adenocarcinoma between 2000 and 2016 in the SEER database were considered for this study. Patients with GC invading the mucosa (T1a) or submucosa (T1b) were selected. However, patients with tumors located at the cardia or esophagogastric junction were excluded. Patients were also excluded based on the following criteria: (1) younger than 60 or older than 90 years old; (2) the clinical or follow-up information was not clear; (3) the survival time was less than one month; (4) death was from diseases other than GC. After applying these criteria, 1911 patients were included for further analyses.

Sex and age of the patient, size and site of the primary tumor, grade, extent of invasion, number of retrieved and metastatic LNs, adjuvant therapy, follow-up duration, and survival status at the last follow-up (SEER cohort: Nov 2018; CMU cohort: Nov 2016) were selected for the current analyses. Tumor, node, and metastasis (TNM) stage was classified according to the eighth edition of the American Joint Committee on Cancer (AJCC) Cancer Staging Manual 1, 17.

### Development and Validation of the Nomogram

After the random distribution, two-thirds of the patients older than 60 years were assigned to the training cohort (n = 1274) and the rest were assigned to the validation cohort (n = 637).

To identify the specific predictors for LN metastasis, a logistic regression model was used to evaluate the correlation between the occurrence of LN metastasis and the following factors in univariate and multivariable analyses: sex, age, primary site and size of the tumor, grade, and T stage (which could be obtained in preoperative examinations). Afterward, based on the logistic regression model results, a nomogram was constructed to assess the risk of LN metastasis. Adjuvant therapy, both preoperatively and postoperatively, is performed with slightly different protocols and screening in different areas, which may affect the effectiveness of the construction and validation of prediction model, especially the current preoperative prediction model. Balachandran et al. also indicated that treatment should be avoided as a covariate in the prediction model unless there were validated data from a randomized clinical trial 18. In the light of these considerations, we did not include neoadjuvant therapy in our prediction model.

The performance of the nomogram was evaluated from the perspective of discrimination and calibration. Discrimination was evaluated by the receiver operating characteristic (ROC) curve and area under the curve (AUC), which reflect the sensitivity, specificity, and accuracy of the model 19, 20. Calibration curves were performed by comparing the predicted risk of LN metastasis from the nomogram with the observed actual incidence 21. We used the bootstrapping (1000 repetitions) method to reduce the bias. An established nomogram was used to calculate the score for each of the patient validation groups for external nomogram validation. Finally, to measure clinical utility, a decision curve analysis (DCA) was conducted by measuring the net benefits for a group of threshold probabilities.

### Statistical Analysis

Disease-specific survival (DSS) was defined as the survival time from resection to death due to GC. The Kaplan-Meier method was applied to calculate DSS and was verified by the log-rank test. The categorical variables were described as counts and proportions. Cox proportional hazards regression models were applied to identify the prognostic factors in univariate and multivariable analyses. The Kruskal-Wallis test was used to confirm the relationship between pN stage and LN metastasis risk groups. The reverse Kaplan-Meier method was used to quantify follow-up 22-24.

Statistical analyses were carried out using R software (version 3.5.3; R Foundation for Statistical Computing, Vienna, Austria) and SPSS (version 23.0; SPSS Inc., Chicago, IL). A two-tailed P-value < 0.05 was considered statistically significant in all analyses.

## Results

### Clinicopathological Characteristics and Survival Analyses

A total of 1911 EGC patients over 60 years old were found for further study, including 1763 from the SEER database and 148 from the CMU database. The demographic and pathological characteristics are illustrated in Table 1. More than half of the patients were male. The median ages of the CMU cohort and the SEER cohort were 69 and 73 years, respectively. The median follow-up times calculated by the reverse Kaplan-Meier method were 103 months for the CMU cohort and 66 months for the SEER cohort. Moreover, the incidence of LN metastasis was 21.6% in the CMU cohort and 19.2% in the SEER cohort. More than 50% of LN metastatic patients had one or two mLNs (N1 stage). Of the patients without mLNs in both cohorts, the distribution of patients with T1a tumors and patients with T1b tumors was similar. However, over 80% (313 of 371) of patients with mLNs were diagnosed with T1b tumors.

**Figure 2:**
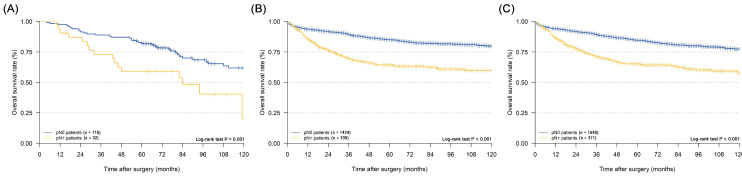
Kaplan-Meier disease-specific survival curves of elderly EGC patients with or without lymph node metastasis: (A) CMU cohort; (B) SEER cohort; (C) merged cohort.

In the survival analyses, we found that EGC patients with mLNs had a significantly poorer prognosis in the merged cohort (Figure 2A, five-year DSS: 84.6% vs. 65.2%, P < 0.001). The same outcomes were confirmed in the SEER cohort and the CMU cohort (Figure 2B and 2C, P < 0.001 and P = 0.001, respectively).

### Univariate and Multivariate Analyses for Predictive Factors

The merged cohort was divided into a training cohort and a validation cohort by a ratio of 2:1. The characteristics are illustrated in Table 2. The training cohort included 1274 patients and was applied to develop predictive factors and construct a nomogram. The validation cohort included 637 patients and was used to validate the capacity of the nomogram.

The univariate and multivariate analyses by the logistic regression model of the training cohort are illustrated in Table 3. The size of the primary tumor, the grade, and the T stage were identified as factors being significantly associated with the occurrence of LN metastasis (all P < 0.05). The significant factors were included in multivariate logistic regression analyses, and all maintained significance (all P < 0.05). Tumors >4 cm [odds ratio (OR) = 4.607, 95% confidence interval (CI): 2.644-8.026] had a significantly higher risk of LN metastasis than smaller tumors. A poorer degree of differentiation also reflected a higher occurrence rate of LN metastasis for EGC patients (P = 0.014). In addition, the risk of LN metastasis in pT1b patients was approximately 3.419 times higher than pT1a patients (OR = 3.419, 95% CI: 2.329-5.019). Furthermore, the size of the primary tumor, the grade, and the T stage were selected to establish the nomogram to predict the risk of LN metastasis.

### Nomogram Development and Validation

**Figure 3:**
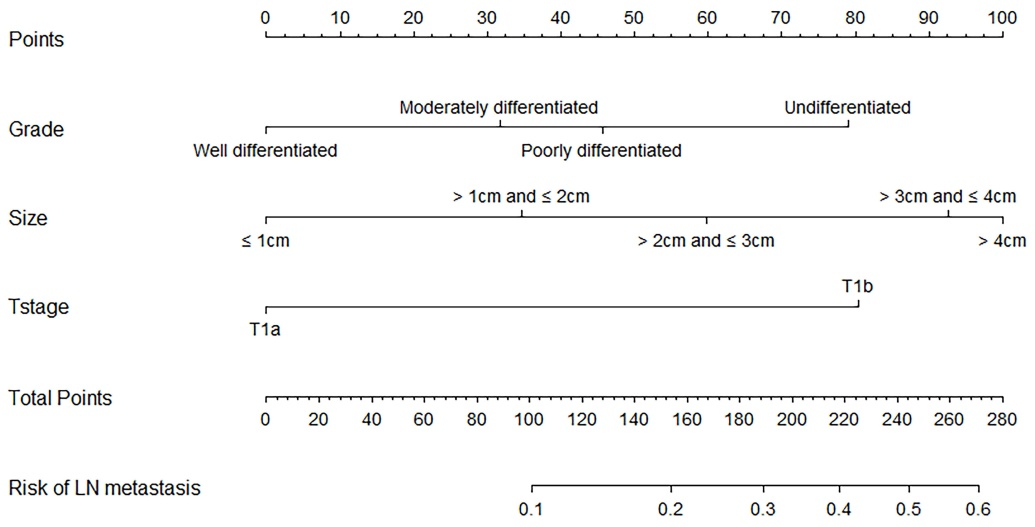
Nomogram predicting lymph node metastasis risk of elderly EGC patients basing on the univariate and multivariate logistic regression models.

**Figure 4:**
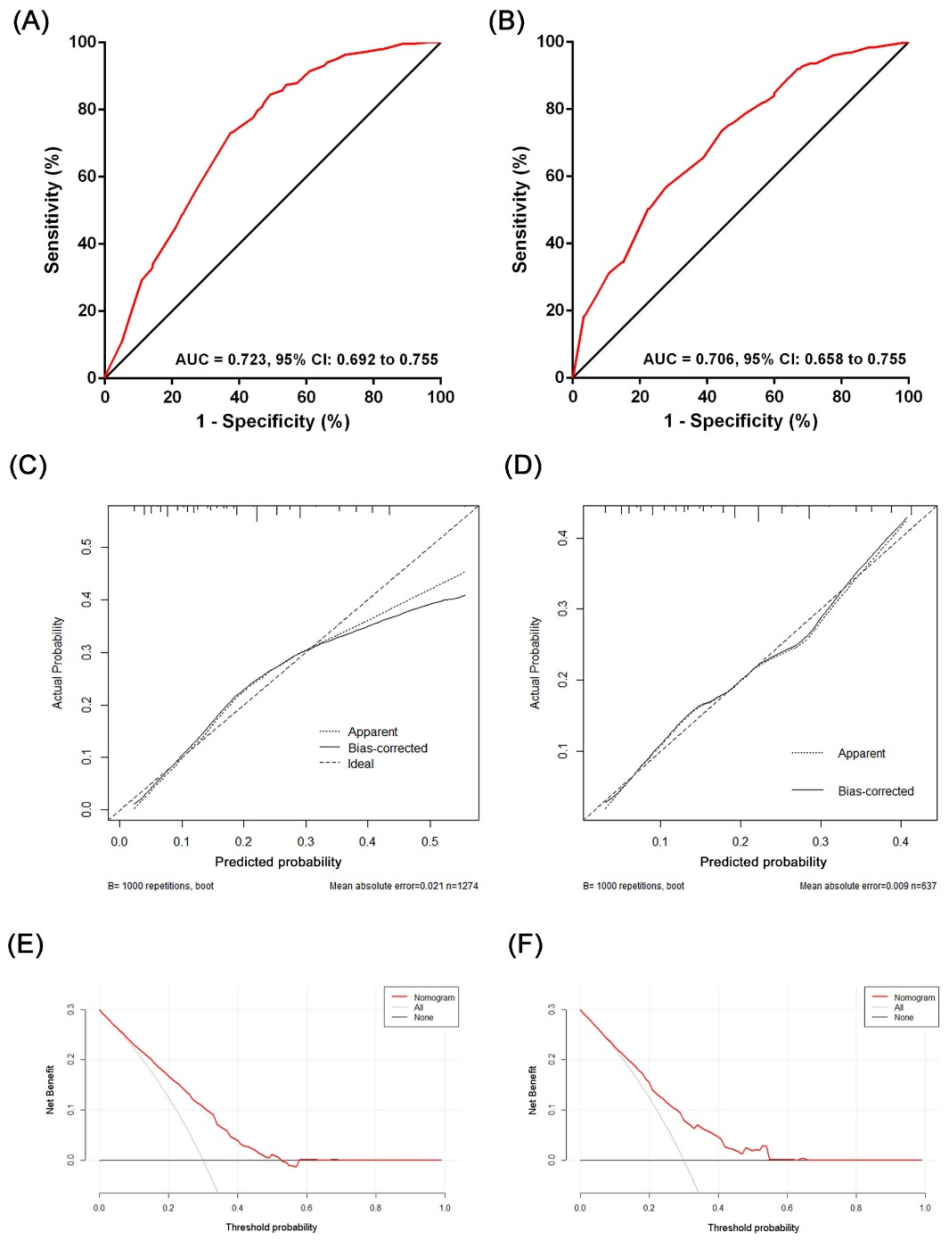
The evaluation of the performance of the nomogram predicting lymph node metastasis in the training cohort and the validation cohort: (A) ROC plot of the nomogram in the training cohort. The AUC was 0.723 (95% CI: 0.692-0.755); (B) ROC plot of the nomogram in the validation cohort. The AUC was 0.706 (95% CI: 0.658-0.755); (C) Calibration plot in the training cohort (1000 repetitions), mean absolute error = 0.021; (D) Calibration plot in the validation cohort (1000 repetitions), mean absolute error = 0.009; (E) The DCA curve of the training cohort; (F) the DCA curve of the validation cohort.

Based on the results of the multivariate analysis, we established a nomogram that could predict the risk of LN metastasis in the training cohort (Figure 3). With this nomogram model, it was easy to obtain an individualized summing score based on selected variables for each patient and estimate the risk of LN metastasis. The AUC in the training cohort for the nomogram to predict LN metastasis was 0.723 (Figure 4A, 95% CI: 0.692-0.755). Discrimination was also validated in the validation cohort and the AUC was 0.706 (Figure 4B, 95% CI: 0.658-0.755). To validate similarities between LN metastasis risk predicted by the nomogram model and actual rates, calibration curves were created in both the training and validation cohorts (Figure 4C, 4D). DCA curves also showed that the nomogram for prognostic prediction had a good ability in clinical utility (Figure 4E and 4F). The results illustrated that the nomogram performed well in predicting LN metastasis risk without significant error in either cohort.

### Survival and Relationship Analyses of Different Risk Levels of LN Metastasis

**Figure 5:**
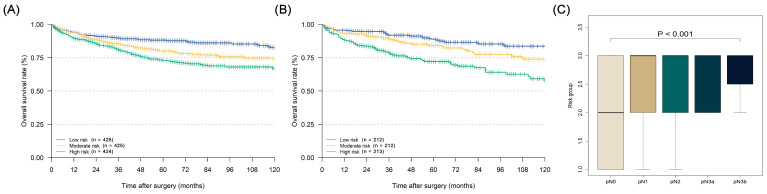
Kaplan-Meier disease-specific survival curves of elderly EGC patients under different degrees of lymph node metastasis risk and the relationship analysis between pN stage and lymph node metastasis risk: (A) Kaplan-Meier curve of training cohort; (B) Kaplan-Meier curve of validation cohort; (C) relationship between pN stage and lymph node metastasis risk under the Kruskal-Wallis test (P < 0.001).

After each patient's individualized summing score was obtained through the nomogram, we divided the patients into three equal groups according to their scores: low-risk group (with a risk of LN metastasis lower than 11.7%), moderate-risk group (with a risk of LN metastasis from 11.7% to 24.8%), and high-risk group (with a risk of LN metastasis over 24.8%). The Kaplan-Meier curves of the three groups in both the training and validation cohorts are illustrated in Figures 5A and 5B (training cohort: five-year DSS: low risk 88.1% vs. moderate risk 80.0% vs. high risk 72.9%, P < 0.001; validation cohort five-year DSS: low risk 89.0% vs. moderate risk 84.3% vs. high risk 72.2%, P < 0.001). In the prognostic factor analyses by the Cox proportional hazards regression model in the whole cohort, we confirmed that the LN metastasis risk was also an independent prognostic factor for EGC patients who were over 60 years old (high-risk group: HR = 1.752, 95% CI: 1.337-2.296; moderate-risk group: HR = 1.400, 95% CI: 1.062-1.848), along with sex, age, pN stage, and the number of examined LNs (Table 4, P < 0.001). Furthermore, in the relationship analysis, patients in higher-risk groups were confirmed to have a more severe pN stage (Figure 5C, P < 0.001).

## Discussion

In the current multicenter study, a prediction nomogram model of LN metastasis—which allowed for the rapid assessment of metastasis risk through preoperative examinations—was established based on a large cohort of elderly patients with EGC. Through our study, we confirmed that the LN metastasis risk assessed by the nomogram was an independent prognostic factor for the current cohort. We thought that the elderly population with EGC under a high or moderate risk of LN metastasis could consider undergoing gastrectomy instead of ESD at the time of initial diagnosis, thus avoiding the trauma caused by a second surgery for some patients.

Nowadays, EMR and ESD have gradually become the optimal treatment choice for EGC patients 3. However, these two methods cannot achieve curative resection for every case on account of the limitations of endoscopic and imageological predictions for LN metastasis 5-7. It was reported that in the Japanese population, LN metastasis was found in 8.4-12.3% of patients who underwent radical surgery after ESD for EGC 9, 25. In the current study, 539 EGC patients from the whole CMU and SEER cohorts before screening for age were diagnosed with LN metastasis, and 371 (68.8%) were elderly patients. Moreover, in EGC with LN metastasis, recurrence was also inevitable. Approximately 2.2%-7.0% of patients with EGC experienced recurrence after gastrectomy 2, 11, 26, 27. In addition, T1 patients with a higher N stage, such as N2 and N3, had a significantly higher recurrence rate (T1N2: 16.4%; T1N3a: 38.5%; T1N3b: 85.7%) 28. In the Western world, although ESD was less widely accepted in its early years due to factors such as technical proficiency, recent studies on ESD in Western centers have shown comparable results to that in Eastern 29-31. In a Western prospective study of 179 EGC underwent ESD, en bloc and R0 resection rates were up to 98.4% and 90.2%, respectively. The bleeding rate was 6% and the perforation rate was 1% 30. In comparison, multicenter studies of ESD for EGC at Eastern institutions have reported similar en bloc resection rates of 92.7-96.1% and R0 resection rates of 82.6-94.5% with bleeding rates of 0.6-9.9% and perforation rates of 3.6-4.7% 31. In a retrospective case-control study including 260 western EGC patients, ESD could significantly reduce the operation time and postoperative hospitalization stay, and prolong 5-year disease-free survival (DFS) (All P < 0.05) 32. However, a retrospective analysis based on the National Cancer Database indicated that endoscopic resection was inferior in terms of prognosis for cT1aN0 and cT1bN0 GC when compared with gastrectomy and suggested that a construction of a subset considering the risk of LN metastasis was beneficial to prolong EGC patients' survival from endoscopic resection 33. With the globalization of ESD and the increasing mastery of ESD technology by doctors in the Western world, the application of ESD in the Western world will gradually become widespread. Therefore, it is also of great significance in the Western world to first assess the risk of LNM in elderly patients with EGC and then select the best treatment to reduce the physical trauma caused by unnecessary secondary surgery.

Therefore, there have been studies aimed at establishing scoring systems to assess the risk of LN metastasis for EGC patients 9, 10, 13. To evaluate whether reoperation would be necessary after ESD, Hatta et al. 13 established a scoring system known as the eCura system to assess the postoperative LN metastasis rate in 15,785 EGC patients with ESD. The system was adopted by the fifth edition of the Japanese Gastric Cancer Treatment Guideline 14. The system included tumor size, tumor depth, lymphatic invasion, venous invasion, and vertical margin. It reported that patients with a low risk had a 2.5% rate of LN metastasis, intermediate risk a 6.7% rate of LN metastasis, and high risk a 22.7% rate of LN metastasis. Furthermore, the five-year DSS differed significantly among the three groups (low risk: 99.6%; intermediate risk: 96%; high risk: 90.1%). The study showed that patients with a low risk were more suitable to undergo ESD without additional treatment. Moreover, the eCura system was a seven-point system. Another 11-point risk-scoring model for LN metastasis was based on 3483 EGC patients and also included five variables: tumor size, tumor depth, histological type, ulcerative findings, and status of lymphovascular invasion 6. Compared to the 11-point system, the advantages of the eCura system were that it was based on multi-center populations and could evaluate LN metastasis and DSS very well. Mu et al. 10 established a nomogram for 872 EGC patients to predict LN metastasis by four variables: lymphovascular invasion, degree of differentiation, tumor size, and extent of invasion. The nomogram was also designed to determine whether additional LN resection was necessary.

The eCura system of others might be suitable for patients who could successfully tolerate a second surgery, but for some elderly or sickly patients, reoperation might put these patients at risk of death not from the EGC itself. It was also reported that there was a higher risk of complications or death from reoperation 34. It was also reported that in multivariate analyses, patients over 50 years with T1b GC had a significantly higher possibility of LN metastasis than younger patients (OR = 2.703, 95% CI: 1.126-6.494, P = 0.026) 35. Therefore, it is necessary to establish an assessment system that can differentiate, at the time of initial diagnosis, elderly patients for whom it would be better to undergo radical surgery than ESD.

At present, the diagnostic methods of EGC rely mainly on endoscopic and pathological biopsy. The size and grade of the tumor can be obtained. To determine the depth of invasion, endoscopic ultrasonography has been confirmed to have high accuracy 36. Thus, according to the former methods, after the initial diagnosis of EGC, the nomogram established in the current study according to the grade, tumor size, and T stage performed well and could rapidly predict the risk of LN metastasis for elderly patients. It is worth mentioning that the grade obtained by local biopsy is not representative of the whole tumor. Therefore, during endoscopic examination, a multi-point biopsy should be conducted on the tumor site when possible. This would improve the accuracy of the grade. Based on the model, the predicted rate of LN metastasis ranged from 2.3% to 55.7% in the whole cohort. In the low-risk, moderate-risk, and high-risk groups, the median rates of LN metastasis were 6.2%, 18.4%, and 35.3%, respectively. Whether to perform ESD or gastrectomy varies in different patients' perceptions, countries, and policies 14, 37, 38. The current study's risk-prediction system could become a reference for clinical decision making after the initial diagnosis of EGC in elderly patients worldwide.

The current study also has several limitations. First, our analyses were based on retrospective data, and the selection principle was based on diagnosis, demographic and pathological characteristics, and other information existing in the CMU and SEER databases. Second, the molecular factors related to tumor malignancy or LN metastasis were not included in the nomogram. Third, the analyses of adjuvant therapy or neoadjuvant therapy are limited due to the lack of detailed chemotherapy-related information in the both databases. If a favorable response is observed and the tumor regresses to ypT1 from neoadjuvant therapy, it is still not considered to be EGC and these patients are not suitable for endoscopic resection. Although this part of patients was not excluded out in the current study, these patients tended to have larger tumor size and lower degree of differentiation than patients initially diagnosed with EGC, thus having a higher probability of being classified as high-risk patients in our model. Since the prognosis of EGC patients is generally optimistic and the recurrence rate is low, applying DFS to analyze clinical data and constructing the nomogram might be more consistent with the actual clinical situations. However, there is no specific information on DFS in the SEER database. For small tumors that are difficult to biopsy at many sites, the representativeness of the diagnostic results of grade would be limited. In this situation, the clinicians' comprehensive and empirical judgment is more important to determine the final treatment of patients. Furthermore, even though we built the training cohort and the validation cohort to evaluate the effectiveness of our model, the external data must still be validated. We will conduct a randomized prospective study including patients receiving neoadjuvant therapy to explore the effectiveness of the model and optimize it.

In conclusion, the nomogram established in the present study could predict the risk of LN metastasis of elderly patients with EGC before ESD or EMR. It is important to minimize the likelihood of reoperation, especially in the elderly population. The value of this nomogram for elderly EGC patients from other institutions will be determined in future studies.

## Figures and Tables

**Figure 1:**
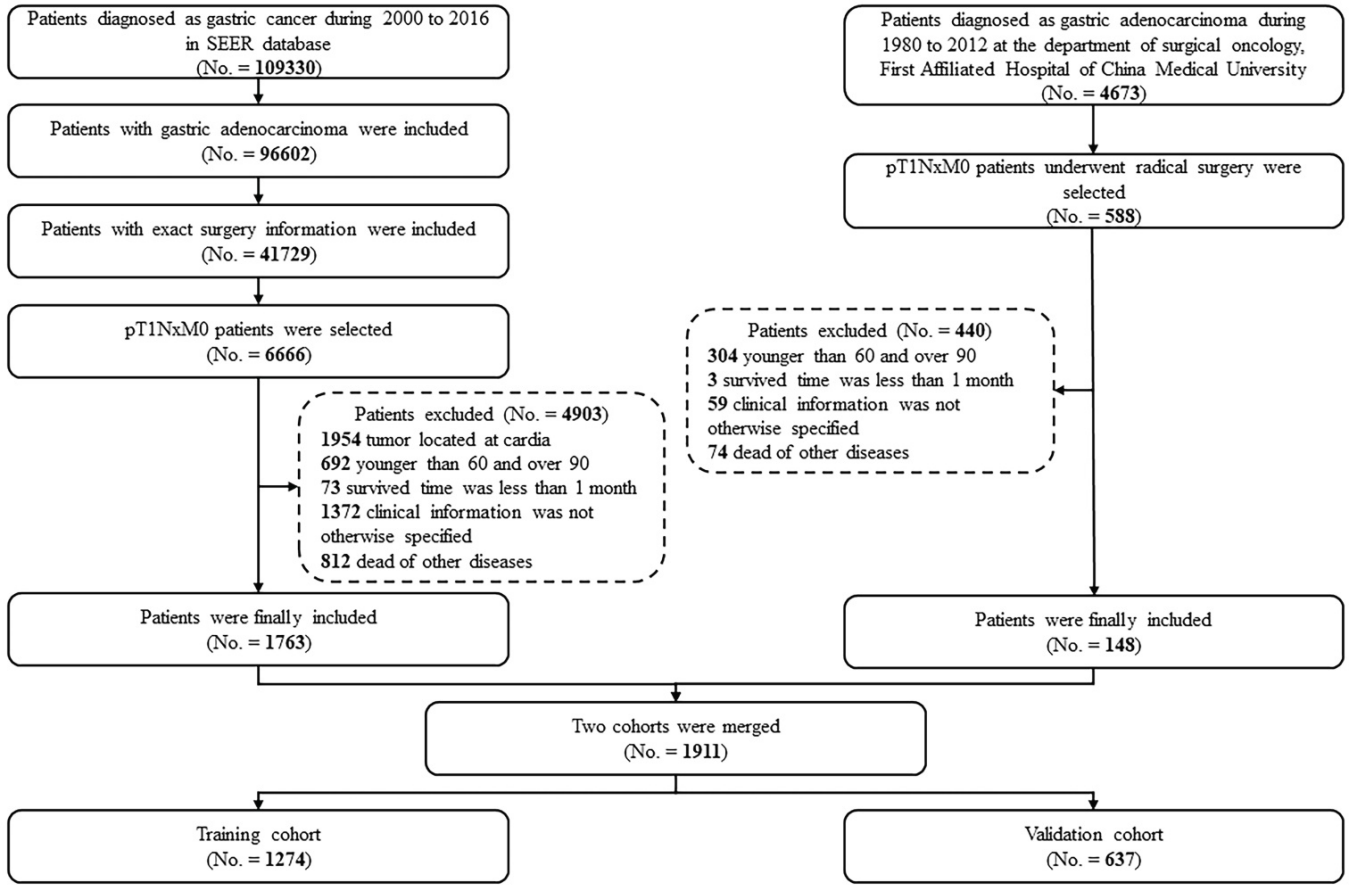
Case screening process for the current analyses from the CMU and SEER database.

**Table 1:** Demographic and pathological characteristics of the 1911 patients in the current study.

Characteristics	CMU cohort (No. = 148)			SEER cohort (No. = 1763)		
	All	N0 (No. = 116, 78.4%)	N+ (No. = 32, 21.6%)	All	N0 (No. = 1424, 80.8%)	N+ (No. = 339, 19.2%)
**Sex**						
Male	116	92 (79.3%)	24 (75.0%)	960	770 (54.1%)	190 (56.0%)
Female	32	24 (20.7%)	8 (25.0%)	803	654 (45.9%)	149 (44.0%)
**Tumor location**						
Upper	10	10 (8.6%)	0 (0.0%)	61	51 (3.6%)	10 (2.9%)
Middle	20	14 (12.1%)	6 (18.8%)	281	233 (16.4%)	48 (14.2%)
Lower	99	78 (67.2%)	21 (65.6%)	808	641 (45.0%)	167 (49.3%)
Mixed	19	14 (12.1%)	5 (15.6%)	613	499 (35.0%)	114 (33.6%)
**Grade**						
Well differentiated	40	28 (24.1%)	12 (37.5%)	264	245 (17.2%)	19 (5.6%)
Moderately differentiated	29	20 (17.2%)	9 (28.1%)	622	512 (36.0%)	110 (32.4%)
Poorly differentiated	75	65 (56.0%)	10 (31.3%)	845	645 (45.3%)	200 (59.0%)
Undifferentiated	4	3 (2.7%)	1 (3.1%)	32	22 (1.5%)	10 (3.0%)
**Size**						
≤1cm	16	16 (13.8%)	0 (0.0%)	399	367 (25.8%)	32 (9.4%)
> 1cm and ≤2cm	52	43 (37.1%)	9 (28.1%)	517	441 (31.0%)	76 (22.4%)
> 2cm and ≤3cm	34	26 (22.4%)	9 (28.1%)	359	280 (19.7%)	79 (23.3%)
> 3cm and ≤4cm	25	17 (14.7%)	8 (25.0%)	214	159 (11.2%)	55 (16.3%)
> 4cm	20	14 (12.1%)	6 (18.8%)	274	177 (12.3%)	97 (28.6%)
**T stage**						
T1a	65	60 (51.7%)	5 (15.6%)	645	592 (41.6%)	53 (15.6%)
T1b	83	56 (48.3%)	27 (84.4%)	1118	832 (58.4%)	286 (84.4%)
**N stage**						
N0	116	116 (100%)	0 (0.0%)	1424	1424 (100%)	0 (0.0%)
N1	19	0 (0.0%)	19 (59.4%)	208	0 (0.0%)	208 (61.4%)
N2	9	0 (0.0%)	9 (28.1%)	98	0 (0.0%)	98 (28.9%)
N3a	4	0 (0.0%)	4 (12.5%)	25	0 (0.0%)	25 (7.4%)
N3b	0	0 (0.0%)	0 (0%)	8	0 (0.0%)	8 (2.3%)
**Examined LNs**						
≤15	61	52 (44.8%)	9 (28.1%)	1043	881 (61.9%)	162 (47.8%)
> 15	87	64 (55.2%)	23 (71.9%)	720	543 (38.1%)	177 (52.2%)
**Adjuvant therapy**						
No/ unknown	139	114 (98.3%)	25 (78.1%)	1514	1359 (95.4%)	155 (45.7%)
Yes	9	2 (1.7%)	7 (21.9%)	249	65 (4.6%)	184 (54.3%)

CMU, China Medical University; SEER, Surveillance, Epidemiology, and End Results; No., number of patients; LNs, lymph nodes.

**Table 2:** Demographic and pathological characteristics of the training cohort and validation cohort in the current study.

Characteristics	Training cohort (No. = 1274)		Validation cohort (No. = 637)	
	N0 (No. = 1028)	N+ (No. = 246)	N0 (No. = 512)	N+ (No. = 125)
**Sex**				
Male	574	140	288	74
Female	454	106	224	51
**Tumor location**				
Upper	38	6	23	4
Middle	159	39	88	15
Lower	482	126	237	62
Mixed	349	75	164	44
**Grade**				
Well differentiated	192	25	81	6
Moderately differentiated	351	79	181	40
Poorly differentiated	465	133	245	77
Undifferentiated	20	9	5	2
**Size**				
≤1cm	256	20	127	12
> 1cm and ≤2cm	328	57	156	28
> 2cm and ≤3cm	203	55	103	33
> 3cm and ≤4cm	106	46	70	17
> 4cm	135	68	56	35
**T stage**				
T1a	433	36	219	22
T1b	595	210	293	103
**N stage**				
N0	1028	0	512	0
N1	0	144	0	83
N2	0	76	0	31
N3a	0	23	0	6
N3b	0	3	0	5
**Examined LNs**				
≤15	626	107	307	64
> 15	402	139	205	61
**Adjuvant therapy**				
No/ unknown	979	122	494	58
Yes	49	124	18	67

No., number of patients; LNs, lymph nodes.

**Table 3:** Univariate and multivariate analyses basing on the logistic regression model in the training cohort.

Characteristics		Univariate analysis			Multivariate analysis		
		RR	95% CI	*P* value	RR	95% CI	*P* value
**Sex**	Male	Ref		0.760			
	Female	0.957	0.723-1.268				
**Age**	(continuous)	0.985	0.966-1.005	0.145			
**Size**	≤ 1cm	Ref		< 0.001	Ref		< 0.001
	> 1cm and ≤ 2cm	2.224	1.303-3.798		1.697	0.981-2.937	
	> 2cm and ≤ 3cm	3.468	2.013-5.974		2.492	1.425-4.359	
	> 3cm and ≤ 4cm	5.555	3.136-9.839		4.121	2.290-7.415	
	> 4cm	6.447	3.756-11.068		4.607	2.644-8.026	
**Grade**	Well differentiated	Ref		0.003	Ref		0.014
	Moderately differentiated	1.729	1.066-2.802		1.624	0.981-2.689	
	Poorly differentiated	2.197	1.388-3.477		2.009	1.240-3.255	
	Undifferentiated	3.456	1.419-8.418		3.346	1.300-8.608	
**Tumor location**	Upper	Ref		0.493			
	Middle	1.553	0.613-3.935				
	Lower	1.656	0.685-4.004				
	Mixed	1.361	0.555-3.336				
**T stage**	T1a	Ref		< 0.001	Ref		< 0.001
	T1b	4.245	2.919-6.174		3.419	2.329-5.019	

RR, risk ratio; CI, confidence interval.

**Table 4:** Univariate and multivariate analyses basing on the Cox proportional hazards regression model in the whole cohort.

Characteristics		Univariate analysis			Multivariate analysis		
		HR	95% CI	*P* value	HR	95% CI	*P* value
**Sex**	Male	Ref		0.014	Ref		**0.002**
	Female	0.774	0.631-0.950		0.722	0.587-0.889	
**Age**	(continuous)	1.058	1.042-1.073	< 0.001	1.060	1.044-1.075	**< 0.001**
**Tumor location**	Upper	Ref		0.076			
	Middle	0.579	0.355-0.942				
	Lower	0.554	0.356-0.863				
	Mixed	0.578	0.368-0.909				
**N stage**	N0	Ref		< 0.001	Ref		**< 0.001**
	N1	2.036	1.562-2.652		1.903	1.420-2.551	
	N2	2.756	1.994-3.808		2.925	2.024-4.228	
	N3a	3.313	1.897-5.787		3.836	2.106-6.987	
	N3b	5.297	2.184-12.847		6.731	2.673-16.949	
**Examined LNs**	≤15	Ref		0.018	Ref		**0.004**
	> 15	0.780	0.634-0.959		0.725	0.584-0.900	
**Adjuvant therapy**	No/ unknown	Ref		0.004	Ref		0.532
	Yes	0.677	0.520-0.881		1.108	0.804-1.527	
**LN metastasis risk**	Low	Ref		< 0.001	Ref		**< 0.001**
	Moderate	1.533	1.166-2.017		1.400	1.062-1.848	
	High	2.283	1.766-2.952		1.752	1.337-2.296	

HR, hazard ratio; CI, confidence interval; LNs, lymph nodes.

## Abbreviations

EGC: early gastric cancer
LN: lymph node
GC: gastric cancer
ESD: endoscopic submucosal dissection
EMR: endoscopic mucosal resection
mLNs: metastasis lymph nodes
JGCA: Japanese Gastric Cancer Association
CMU: China Medical University
SEER: Surveillance, Epidemiology, and End Results
TNM: tumor, node, and metastasis
AJCC: American Joint Committee on Cancer
ROC: receiver operating characteristic
AUC: area under the curve
DCA: decision curve analysis
DSS: disease-specific survival
OR: odds ratio
CI: confidence interval
DFS: disease-free survival
